# Little Causes of Death

**Published:** 1871-06

**Authors:** 


### LITTLE CAUSES OF DEATH.

There are times when a man is in such vigorous health, that it almost seems as if nothing could hurt him.

A young lady alighted from her carriage in the Central Park and stood upon the damp grass for half an hour, listening to the music of the band; the feet became cold, a chill struck through her system, and she died in a few days of a disease in the throat.

Recently a young lady from New York was visiting some of the interesting places in Rome; becoming weary, she sat on a stone bench for a few moments, and has been paralyzed from her waist down, ever since.

A person walks on a summer's day to some public exhibition or picture gallery; it feels refreshingly cool on first entrance, time passes unawares, when all at once an ugly chill runs down the back, and next day there is a cold which is to worry and annoy for weeks, if not months. Very many, especially delicate ladies, "get their death" in this way, in visiting the picture galleries and public buildings of the Old World; the precaution should be taken always on such occasions to have an extra shawl along, even in the hottest days of summer, to be thrown over the shoulders, and if the gloves are kept on, it is a great aid in preventing the system from cooling off too rapidly.

It is a simple and a very little thing to take a bath. A young lawyer took a bath some years ago, in this city; he stayed in too long, became chilled, and was a cripple all his days, for forty years afterwards.

Many a person has been found dead in the bath-tub, from attempting to enjoy that luxury too soon after eating. Never bathe, hot or cold, on a full stomach; the most perfectly safe time for taking any kind of bath is before breakfast, for then there is the least danger that the system is overheated, and then too it has the most vigor, the greatest power of reaction, the greatest ability of resisting the influence of cold.

It is usual for sea-bathings to be taken in the middle of the forenoon or afternoon; but considering the hot sun, sending its fiercest rays on the head, it would be wiser and safer to select the early morning.

